# Research on a Regional Availability Evaluation Model for Road-Area High-Entropy Energy Based on Synergy Factors

**DOI:** 10.3390/e28060715

**Published:** 2026-06-22

**Authors:** Juexiao Chen, Yinlin He, Lei Shi, Yihao Tao

**Affiliations:** College of Automotive and Energy Engineering, Tongji University, Shanghai 201804, China; chenjuexiao@tongji.edu.cn (J.C.);

**Keywords:** road-area high-entropy energy, regional availability, synergy factors, best alignment similarity, spatial consistency

## Abstract

To address the challenge of quantifying multi-unit synergy effects in road-area high-entropy energy systems, this paper proposes a regional availability evaluation model based on synergy factors. In the revised model, regional availability is decomposed into the product of a capacity-weighted health baseline (capacity-weighted mean unit availability), weighted temporal synergy, and weighted spatial consistency coefficient. Capacity weights and pairwise coupling coefficients are introduced to extend the model from equal-capacity isomorphic units to heterogeneous road-area energy units. Simulation results demonstrate that the model can distinguish different synergy levels, and parameter sensitivity analysis verifies its robustness. An open-data-based quasi-real verification using Caltrans PeMS traffic records further shows that the model can process measured time-series inputs. The proposed model provides a theoretical basis for the regional-level operation evaluation of road-area energy systems.

## 1. Introduction

Road-area high-entropy energy systems represent a class of multi-energy coupling networks distributed along highways, encompassing typical scenarios such as microgrids in expressway service areas and tunnel lighting and ventilation systems [1]. These operational architectures are characterized by a massive number of interconnected units, highly diverse energy forms, and strongly coupled operational states. Consequently, the overall performance of such systems depends not merely on the individual availability of each unit but fundamentally relies on the synergistic coordination among multiple units.

In the field of distributed energy systems, the previous literature has systematically reviewed applications ranging from individual buildings to broad regional levels. Studies indicate that evaluating distributed energy networks requires comprehensive consideration of multidimensional indicators, spanning energy, environmental, and economic domains. Furthermore, as the penetration rate of renewable energy steadily climbs, system flexibility emerges as a critical necessity, which must be achieved through the coordinated operation of multiple energy carriers [2]. The literature [3] points out that accurately describing the decentralized energy output characteristics and effectively integrating them into the power grid remain paramount to maintaining system stability.

Our research team previously established and validated a unit-level availability evaluation model, which defines unit availability as the product of its output capacity factor and behavioral orderliness factor. The former metric reflects the ratio of the actual equipment output to its rated capacity, while the latter characterizes the fluctuation and randomness inherent in the output. Nevertheless, a purely unit-level evaluation framework cannot resolve critical questions: How should the overall availability of a region be evaluated when units have different rated capacities or even different energy forms? Moreover, how should regional availability be properly discounted when some units perform well individually but exhibit poor synergistic coordination as a collective?

Within road-area distributed high-entropy energy systems, the output of individual energy units exhibits pronounced spatiotemporal correlation mixed with strong stochasticity. When multiple units are connected to a unified regional network, their contributions to regional availability are not necessarily equal; a unit with a larger rated or equivalent service capacity should have a larger influence on the regional health baseline. Meanwhile, their output fluctuations may superimpose, cancel out, or resonate, significantly affecting the region’s overall power support capacity delivered to the grid [4]. Traditional reliability models often assume independent component operation, leading to a simplified summation of individual capabilities. Such methodologies may fail to capture the effectiveness generated by collective collaboration, meaning evaluations based solely on individual unit availability may inaccurately estimate the region’s actual dispatchable capacity.

To bridge this methodological gap, this paper introduces a regional availability evaluation model tailored for road-area high-entropy energy systems based on synergy factors. The revised framework retains the three dimensions of structural baseline, temporal synergy, and spatial synergy, and further introduces capacity weights and pairwise coupling coefficients. The model is validated through numerical case studies, parameter sensitivity analysis, and an open-data-based PeMS verification.

It is necessary to clarify that this study focuses strictly on the following boundary conditions and fundamental assumptions:

1. The considered regional system is not restricted to equal-capacity isomorphic units. For units belonging to different energy forms, their outputs should first be converted into comparable normalized sequences, such as equivalent electric power, available service capacity, or exergy-equivalent contribution. Pairwise coupling coefficients are then used to represent the strength of physical, operational, or dispatchable relationships between heterogeneous units. The equal-capacity isomorphic case is retained as a special case of the generalized formulation.
2. This paper employs best alignment similarity based on cross-correlation analysis to quantify the synergistic potential among constituent units. This specific method operates under the assumption that an identifiable time delay relationship exists among unit output sequences, and this delay strictly falls within an engineering-allowable range. For pairs exhibiting completely random outputs devoid of any correlation, their calculated best alignment similarity inherently approaches 0, ensuring the model remains mathematically sound.
3. The effective practical application of this model necessitates robust output data support. Specifically, the calculation of best alignment similarity requires the length of each unit’s output sequence to satisfy specific statistical conditions to guarantee the reliability of the cross-correlation analysis. Data preprocessing is mandatory for systems suffering from missing data or insufficient sampling frequencies.
4. The model utilizes a sliding time window mechanism to accomplish real-time availability evaluation. By adopting the current moment as a functional reference point, a fixed span of historical data serves as the evaluation window to compute localized regional availability. As time predictably progresses, the continuous sliding of this window yields a dynamic time series of regional availability. The careful selection of window length requires balancing real-time operational sensitivity against analytical stability: windows that are too short are easily perturbed by transient transients, whereas overly extended windows precipitate lagging responses.

These explicit boundary conditions clearly demarcate the applicable domain of the proposed model while simultaneously illuminating viable trajectories for subsequent research extensions.

## 2. Regional Availability Model

### 2.1. Model Framework

Consider a road-area high-entropy energy region composed of N energy units, where the standalone availability of unit i is defined as $A_{i}$ and its rated or equivalent service capacity is defined as $P_{i}^{rated}$. To account for different unit contributions, the capacity weight is defined as $w_{i}=\frac{P_{i}^{rated}}{\sum_{j=1}^{N}P_{j}^{rated}}$. For heterogeneous units, $\gamma_{ij}\in \left[0,\;1\right]$ denotes the pairwise coupling coefficient between units i and j. The regional-level availability, denoted as $A_{region}$, comprehensively encapsulates the weighted structural health level of all units, the matching degree of unit outputs across the temporal dimension, and the corresponding spatial uniformity of this temporal matching. This paper establishes a generalized regional availability model structured as a three-factor mathematical product, expressed in Equation (1):

(1) $$A_{region}=B_{w}\cdot S_{w}\left(t\right)\cdot C_{w}$$

where

$B_{w}$ represents the capacity-weighted health baseline, which reflects the fundamental functional health of the region while considering differences in rated or equivalent service capacity among units.

$S_{w}\left(t\right)$ signifies the weighted regional temporal synergy, mathematically anchored by the best alignment similarity between unit output sequences and the pairwise coupling weights of heterogeneous units.

$C_{w}$ acts as the weighted spatial synergy consistency coefficient, designed to represent the spatial uniformity of temporal synergistic effects after considering the relative importance of different unit pairs.

Because all three foundational factors are normalized to the interval [0, 1], it naturally follows that $A_{region}$ also belongs to [0, 1]. The generalized model relaxes the equal-contribution assumption embedded in the original homogeneous setting and enables a more realistic reflection of cluster availability under spatiotemporal coupling constraints.

### 2.2. Calculation of the Capacity-Weighted Health Baseline

The defined health baseline quantitatively gauges the static structural output capacity of the modeled region. Instead of using an arithmetic mean, the revised model defines the health baseline as the weighted mean of all individual unit availabilities, as detailed in Equation (2):

(2) $$w_{i}=\frac{P_{i}^{rated}}{\sum_{j=1}^{N}P_{j}^{rated}},B_{w}=\overset{N}{\underset{i=1}{\sum }}w_{i}\;A_{i}$$

When all units have identical rated capacities, $w_{i}=\frac{1}{N}$ and Equation (2) degenerates into the arithmetic mean used in the homogeneous equal-capacity setting. In practical road-area high-entropy energy systems, however, units with larger rated or equivalent capacity contribute more to regional support capability and should therefore have higher weight. If the capacity-weighted structural baseline $B_{w}$ itself remains low, the regional availability will be restricted regardless of how strong the accompanying synergistic effects might be. This treatment is consistent with the relationship between system availability and component reliability in traditional engineering disciplines [5].

### 2.3. Best Alignment Similarity

Within the context of geographically scattered road-area energy systems, the specific peak values of physical excitations received by discrete units naturally exhibit inherent temporal lags, conventionally known as phase differences. If engineers merely adopt a zero-delay Pearson correlation coefficient, the network’s latent spatiotemporal complementary capacities will be severely underestimated. Literature detailing the intricate complementarity of wind and solar energy explicitly stresses that cross-correlation analysis incorporating time-shift mechanics evaluates spatiotemporal dynamics with superior accuracy [6]. This analytical logic flawlessly applies to the isomorphic energy units discussed herein; whenever inherent phase delays exist across physical outputs, a carefully shifted similarity metric guarantees a more genuine reflection of true synergistic potential.

To capture this, we introduce the best alignment similarity, $\rho_{ij}$, to accurately measure the synergy between unit i and unit j. Assume the normalized operational output sequences of these units are $P_{i}\left[n\right]\;$ and $P_{j}\left[n\right]$ (*n* = 1, 2, …, T), where T signifies the total number of sampling instances during the defined evaluation period. Within the maximum discrete time delay points K structurally permitted by the engineering environment, the system algorithmically traverses all integer time shifts. Given the realistic possibility of bidirectional excitation scenarios—such as opposing highway traffic flows or active tidal lanes—both phase-leading and phase-lagging situations must be analyzed; hence, the respective time shift spans $k\in \left[-K,+K\right]$. As the magnitude of k expands, the physical overlapping length $T-\left|k\right|$ between the matched sequences predictably shrinks. To aggressively protect the statistical reliability governing the correlation coefficient, this overlapped intersection must contain a robust density of data points. Adhering to strict signal processing reliability standards [7], the model mandates that K≪T, typically enforcing K≤0.1T.

We systematically calculate the Pearson correlation coefficient $r_{ij}\left(k\right)\in \left[-1,1\right]$ for the adjusted sequences, isolating the maximum absolute correlation value to serve as the definitive best alignment similarity, outlined in Equation (3):

(3) $$\rho_{ij}=\underset{\left(k\in \left[-K,+K\right]\right)}{\max}\left|r_{ij}\left(k\right)\right|$$

The defining boundary for the maximum discrete time delay points K is collectively determined by the specific unit spacing d, the minimum functional vehicle speed $v_{min}$, and a designated safety factor α, per Equation (4):

(4) $$K=\alpha \cdot \frac{d}{\left(v_{min}\cdot \Delta t\right)}$$

Here, Δt represents the utilized sampling time interval. The integral safety factor α strategically neutralizes stochastic uncertainty factors, including sudden vehicle speed fluctuations and baseline measurement errors. In practical traffic flow conditions, vehicle speeds are not rigidly constant but follow randomized distributions across specific ranges [8]; therefore, introducing mathematical margins atop theoretical calculations becomes essential to adequately cover genuine phase discrepancies. Drawing heavily from the established empirical ranges of margin coefficients applied in civil engineering practices [9], this paper formally adopts $\alpha \in \left[1.2,\;1.5\right]$.

### 2.4. Regional Temporal Synergy

Following structural evaluation, we construct an N×N best alignment similarity matrix R, where its contained elements $\rho_{ij}$ satisfy self-similarity and symmetry. To extend the model to heterogeneous units, a pairwise influence coefficient $\eta_{ij}$ is introduced. In the most general form, $\eta_{ij}$ can be determined by rated-capacity weights, physical topology, dispatch coupling, or expert-defined coupling coefficients; a practical specification is $\eta_{ij}=w_{i}\;w_{j}\;\gamma_{ij}$. The weighted average synergistic potential is then calculated as Equation (5):

(5) $$\eta_{ij}=w_{i}\;w_{j}\;\gamma_{ij},\;\;\rho_{w}=\frac{\sum_{i=1}^{N-1}\sum_{j=i+1}^{N}\eta_{ij}\;\rho_{ij}}{\sum_{i=1}^{N-1}\sum_{j=i+1}^{N}\eta_{ij}}$$

The derived variable $\rho_{w}$ reflects the weighted mean synergistic potential connecting all coupled unit pairs within the evaluated region, constrained to a value range of [0, 1]. When all capacities are equal and $\gamma_{ij}$ = 1 for all unit pairs, Equation (5) degenerates into the original arithmetic pairwise average.

Notably, synergistic enhancements in high-entropy energy networks may exhibit nonlinear behavior. In low-synergy operating zones, basic coordination or dispatching can often produce a clear improvement, whereas in high-synergy zones, the marginal gain decreases because of physical constraints such as meteorological consistency or traffic boundary conditions. This diminishing-return behavior is consistent with complex-system synergy theory [10], and studies on wind power aggregation [11] show that smoothing efficiency decreases as aggregation scale expands.

To model this nonlinear behavior, we utilize an exponential saturation function to map the weighted average synergistic potential $\rho_{w}$ directly onto the regional temporal synergy domain, as defined in Equation (6):

(6) $$S_{w}\left(t\right)=1-exp\left(-\beta \;\rho_{w}\right),\;\;\beta \in \left[2.5,3.5\right]$$

Within the operational domain $\rho_{w}$ in [0, 1], this defined function is monotonically increasing while exhibiting steep initial growth followed by gradual saturation. The parameter β functions as the synergy sensitivity coefficient governing the curve’s overall steepness. A larger β denotes that the system responds more sensitively to potential enhancements; conversely, a smaller β indicates a flatter synergy response.

**Figure 1:**
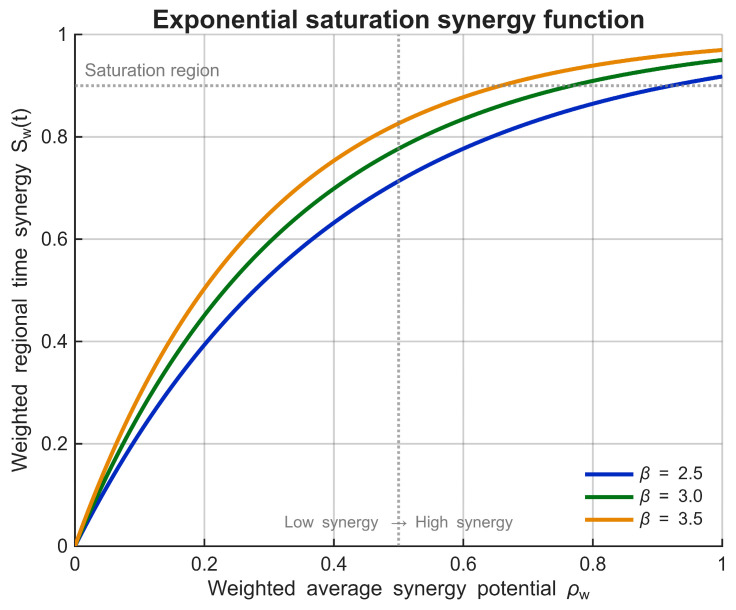
Exponential saturation synergy function curve.

When specifically tailoring the model to accommodate road-area high-entropy architectures, the configuration of β should satisfy operational criteria. Low-synergy regions ($\rho_{w}$ spanning 0.3 to 0.5) should show clear synergistic enhancements through elementary dispatching, while high-synergy zones should appropriately reflect deceleration. The curve family mapped by β in [2.5, 3.5] fulfills both operational requirements. This selected range remarkably mirrors optimized system synergy designs generated through advanced deep-learning frameworks documented in the literature [12]. Moving toward practical deployment, β can be recalibrated by minimizing prediction errors derived from historical logs. The corresponding curve family is shown in Figure 1.

### 2.5. Spatial Synergy Consistency Coefficient

A theoretically high average synergistic potential does not necessarily guarantee a stable regional state, because synergistic properties may be concentrated within a small number of unit pairs. Under such conditions, several specific links may show high structural similarity while many other links remain weakly coordinated. If those high-performance pairs are affected by localized faults or sudden environmental shifts, the region’s aggregate synergy may decrease markedly. Therefore, relying only on the averaged potential metric is insufficient; a spatial synergy consistency coefficient is introduced to measure distribution uniformity.

Defining the weighted synergistic potential set as $\Gamma_{w}$ = {($\rho_{ij}$, $\eta_{ij}$) | i < j}, we compute its weighted mean and weighted standard deviation via Equations (7) and (8):

(7) $$\mu_{w}=\frac{\sum_{i=1}^{N-1}\sum_{j=i+1}^{N}\eta_{ij}\;\rho_{ij}}{\sum_{i=1}^{N-1}\sum_{j=i+1}^{N}\eta_{ij}}$$

(8) $$\sigma_{w}=\sqrt{\frac{\sum_{i=1}^{N-1}\sum_{j=i+1}^{N}\eta_{ij}\;{\left(\rho_{ij}-\mu_{w}\right)}^{2}}{\sum_{i=1}^{N-1}\sum_{j=i+1}^{N}\eta_{ij}}}$$

To quantify the distribution uniformity, the coefficient of variation (CV) is used, as detailed in Equation (9):

(9) $$CV_{w}=\frac{\sigma_{w}}{\mu_{w}}$$

This statistical indicator measures fluctuation amplitude relative to the mean baseline, avoiding the limitation of judging fluctuation only from standard deviation. For example, the same standard deviation may be acceptable when the mean similarity is high, but it may indicate a more unstable state when the mean similarity is low.

Nonetheless, the coefficient’s natural mathematical range makes it structurally inconvenient to insert directly into availability calculations as a standard multiplier. Accordingly, a linear transformation normalizes $CV_{w}$ into the weighted spatial synergy consistency coefficient $C_{w}$, as established in Equation (10):

(10) $$C_{w}=\frac{\mu_{w}}{\mu_{w}+\sigma_{w}}$$

Although alternative mathematical transformations could be used to compress the coefficient into (0, 1], this paper adopts the simple linear variant because it satisfies the following functional criteria:

1. Monotonicity: The selected transformation is monotonically decreasing with respect to the coefficient of variation, ensuring that larger relative dispersion leads to lower spatial consistency.
2. Adaptive behavior: The function decreases more sharply when the mean is low, reflecting the evaluation requirement that unevenness should be penalized more strongly in weak-synergy regions.

Equation (10) enforces that $C_{w}$ = 1 whenever $\sigma_{w}$ = 0, while driving $C_{w}$ toward 0 in extreme cases where either $\mu_{w}$ approaches 0 or $\sigma_{w}$ becomes very large. The built-in adaptive behavior means that identical standard deviations translate into higher consistency outputs when the mean similarity is high, and lower consistency outputs when the mean similarity is low.

The mathematical rationale for Equation (10) is supported by energy system reliability studies. Literature [13] indicates that ignoring the spatiotemporal correlations of dispersed renewable assets can lead to biased reliability modeling, and architectures dependent on isolated key components may be vulnerable to random failures. This aligns with the present model’s focus on the uniformity of the synergy distribution itself. Assessing structural integrity purely from averaged potential may obscure network fragility; therefore, standard deviation is also integrated.

When evaluating resource quality across renewable energy systems, external studies [14] argue that availability, represented by the statistical mean, and variability, represented by the standard deviation, should be analyzed simultaneously. This paradigm is applicable to the spatial distribution of synergy: the combination of synergy magnitude and dispersion controls the region’s overall structural robustness. Networks supported by only a few high-performing pairs remain vulnerable, whereas driving $C_{w}$ toward 1 indicates that synergy is distributed more uniformly across the region.

By mathematically synthesizing Equations (1)–(10), the finalized generalized regional-level availability evaluation model culminates in Equation (11):

(11) $$A_{region}=(\overset{N}{\underset{i=1}{\sum }}w_{i}\;A_{i})\cdot [1-exp\left(-\beta \;\rho_{w}\right)]\cdot [\frac{\mu_{w}}{\mu_{w}+\sigma_{w}}]$$

## 3. Numerical and Quasi-Real Engineering Verification

To verify the effectiveness of the proposed regional availability evaluation model, this section designs multiple simulation scenarios encompassing various synergy levels and boundary conditions, through which the behavioral characteristics and parameter sensitivity of the model are analyzed via numerical experiments. All computations are implemented within a Python 3.13 environment.

### 3.1. Numerical Scenario Design

We define a regional road-area high-entropy network composed of 8 energy units (N = 8), using an adjacent physical unit spacing of d = 10 m, utilizing a sampling interval of Δt = 0.1 s, and enforcing an evaluation sequence length spanning T = 1000 iterations. To reflect capacity heterogeneity, the rated or equivalent service capacities of the eight units are set as [60, 180, 80, 140, 200, 70, 160, 110] kW-equivalent, respectively. The individual structural availabilities mapping to each unit ($A_{i}$) are constrained within [0.85, 0.95] and are listed together with capacity weights in Table 1. These standalone metrics remain fixed across all testing suites to ensure that recorded volatility within the final regional evaluation stems from shifting synergistic interactions rather than baseline alterations.

To adequately mirror real-world operational profiles standard to roadway configurations, the subsequent three targeted archetypal scenarios are structured:

Scenario S1 (Strong Synergy): This operational profile simulates stable traffic conditions with nearly constant driving velocities. Unit outputs are generated from a common reference wave with a deterministic positional delay calculated as $\tau_{i}$ = $x_{i}$/v, where the vehicle velocity is fixed at v = 20 m/s.

Within highway networks, asset outputs usually contain both deterministic and stochastic components. Deterministic components may include daily traffic cycles, diurnal solar variation, or periodic piezoelectric pulses, while stochastic components may arise from speed variation, vehicle mass differences, and local weather fluctuation. Therefore, a smooth sine wave combined with Gaussian white noise at a 20 dB signal-to-noise ratio is used to simulate this mixed output pattern.

Scenario S2 (Medium Synergy): This profile represents more variable traffic conditions. Vehicle speeds are randomly distributed within [5, 30] m/s, and vehicle arrivals are modeled using a Poisson process with an arrival rate of lambda = 0.5 vehicles/second. Partial temporal correlation still exists across the network, but the delay relationship is no longer fixed. Random speed and vehicle-mass factors are therefore added to the generated output signal.

Scenario S3 (Weak Synergy): This profile represents a no-correlation boundary case. Each unit output is generated independently as Gaussian white noise with mean 0.5 and standard deviation 0.2, so the pairwise synergistic potential is expected to be low.

**Figure 2:**
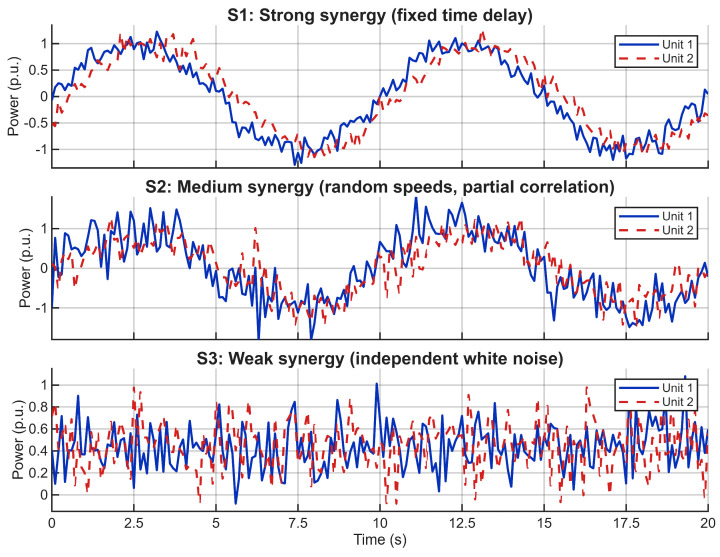
Typical output power waveforms of the three scenarios (segments).

Figure 2 illustrates the typical output power waveforms for two specific units across the three scenarios during the initial 20 s, from which the synergistic characteristics of each scenario can be intuitively observed. In Scenario S1, the waveforms of the two units are highly similar in shape, with Unit 2 exhibiting a fixed time delay of approximately 0.5 s (corresponding to a vehicle speed of 20 m/s) relative to Unit 1. This phenomenon indicates that a clear and predictable time-lag relationship exists between units under stable traffic flow, thereby signifying high synergistic potential. Although the waveforms in Scenario S2 retain partial common trends, the time-delay relationships remain unfixed and are superimposed with strong random perturbations, which reflects a mixed synergistic state resulting from the random distribution of vehicle speeds and the stochasticity of vehicle arrivals in actual traffic flows. In Scenario S3, the waveforms are entirely independent with no discernible correlation, effectively simulating the absence of synergy between units under conditions of extreme randomness.

### 3.2. Availability Calculation

Implementing Equation (2), the capacity-weighted health baseline is computed as follows:

(12) $$B_{w}=\overset{8}{\underset{i=1}{\sum }}w_{i}\;A_{i}=0.897$$

Before processing matrix similarities, bounding the discrete delay ceiling K is mandatory. Incorporating the known parameters, adjacent physical unit spacing d = 10 m, $v_{min}$ = 5 m/s, Δt = 0.1 s, and α=1.3 into Equation (4):

(13) $$K=1.3\times \frac{10}{5\times 0.1}=26$$

By validating that K/T = 26/1000 = 0.026 < 0.1, the statistical overlap requirement is satisfied.

**Figure 3:**
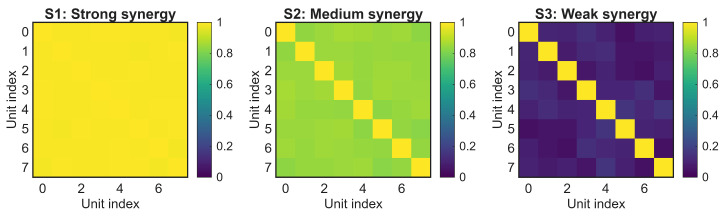
Heatmaps of the best alignment similarity matrix for the three scenarios.

The best alignment similarity matrix for each scenario is calculated according to Equation (3). Figure 3 presents these similarity matrices in the form of heatmaps, where colors closer to yellow indicate higher similarity, while those shifting toward purple represent lower similarity.

The matrix for Scenario S1 predominantly exhibits a bright yellow hue; as indicated in Table 2, $\mu_{w}$ = 0.998. This demonstrates that under the ideal condition of fixed time delays, all unit pairs possess high best alignment similarity, corresponding to strong synergy. The matrix for Scenario S2 is primarily yellow-green, with similarities clustered between 0.84 and 0.85 ($\mu_{w}$ = 0.848). This reflects a clear common trend among units, while random perturbations reduce similarity relative to the ideal scenario. Finally, the matrix for Scenario S3 is dominated by dark purple, featuring generally low similarities ($\mu_{w}$ = 0.083) that are randomly distributed, which corresponds to the weak-synergy condition.

Utilizing Equation (5), the weighted average synergistic potentials $\rho_{w}$ are calculated as 0.998, 0.848, and 0.083, respectively. By substituting these values into Equation (6) and setting β = 3.0, the regional temporal synergy $S_{w}\left(t\right)$ is determined to be 0.950, 0.922, and 0.221 for each respective scenario. These quantitative results are consistent with the intuitive visual characteristics of the heatmaps and show that the model can quantify varying levels of synergy.

For the present numerical scenarios, all generated output sequences are normalized into a comparable service dimension. To preserve comparability with the previous simulation and isolate the role of capacity weighting in the health baseline, pairwise influence coefficients are set as $\eta_{ij}$ = 1 for all unit pairs. Under this setting, the weighted mean $\mu_{w}$ and weighted standard deviation $\sigma_{w}$ degenerate into the original unweighted values and are computed via Equations (7) and (8). Subsequently, the weighted spatial synergy consistency coefficient $C_{w}$ is derived using Equation (10), with the finalized results detailed in Table 2.

Collating all functional factors finalizes the overarching regional availability, detailed in Table 3.

### 3.3. Numerical Result Analysis and Sensitivity Analysis

As can be observed from Table 3, the regional availability across the three scenarios exhibits a distinct gradient. The same capacity-weighted health baseline is used across all scenarios, indicating that the differences in regional availability are primarily caused by synergistic effects.

Comparing Table 1 and Table 2 reveals that the strong synergy scenario not only possesses a high weighted average synergistic potential but also features a highly uniform synergy distribution. Consequently, both the temporal synergy $S_{w}\left(t\right)$ and the spatial consistency $C_{w}$ approach 1. Although the weighted average synergistic potential of the medium synergy scenario is slightly lower than that of the strong synergy scenario, its synergy distribution remains similarly uniform. Therefore, the $C_{w}$ value remains high, resulting in a final regional availability marginally lower than that of the strong synergy scenario. The weak synergy scenario exhibits a low mean alongside a relatively large dispersion, causing $C_{w}$ to drop. This demonstrates the adaptive nature of $C_{w}$: it becomes more sensitive to unevenness when the mean is low.

The scenario analysis should be interpreted as a comparison among predefined coordination states rather than as an expected engineering improvement ratio. In the present results, the strong and medium cases differ by (0.847 − 0.822)/0.822 = 3.0%, while the weak-synergy case is much lower. This pattern indicates that regional availability is sensitive to the transition from disorder to coordinated operation, but has a smaller marginal change once synergy is already high.

**Figure 4:**
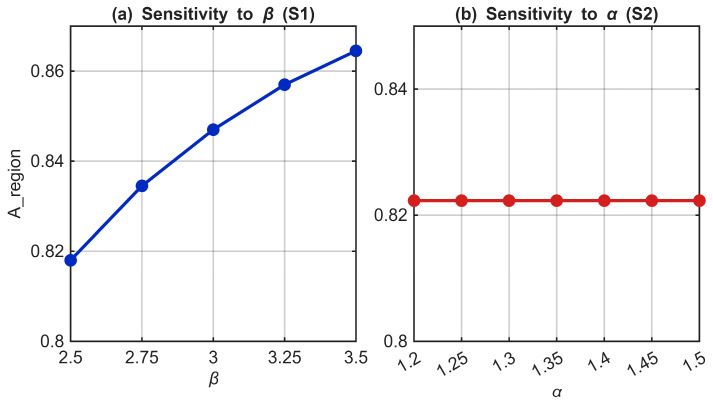
Parameter sensitivity analysis: (**a**) impact of *β* on regional availability; (**b**) impact of the safety factor *α* on regional availability.

To verify the robustness of the model against key parameters, the following sensitivity analysis is conducted. Fixing other parameters and taking β in [2.5, 3.5], $S_{w}\left(t\right)$ and $A_{region}$ for Scenario S1 are calculated, with the results shown in Figure 4a. As β increases from 2.5 to 3.5, $A_{region}$ increases from 0.818 to 0.865, representing a variation margin of approximately 5.7%. This indicates that the model possesses good robustness within the recommended range of β.

Furthermore, taking α = 1.2, 1.3, 1.4, and 1.5, which correspond to K = 24, 26, 28, and 30, respectively, $S_{w}\left(t\right)$ and $A_{region}$ for Scenario S2 are calculated, with the results presented in Figure 4b. The variation in α has a minimal impact on the results, with the fluctuation of $A_{region}$ remaining below 2%, illustrating that the model is insensitive to the safety factor within the recommended range.

Through the simulation scenarios and parameter sensitivity analysis, the characteristics of the proposed regional availability are verified as follows:

1. It can effectively distinguish different levels of synergy, and the variations in regional availability are primarily contributed by synergy factors;
2. The temporal synergy and the spatial consistency coefficient function synergistically to mutually reflect the strength and robustness of regional synergy;
3. The results exhibit good robustness when the key parameters *β* and *α* vary within their recommended ranges.

### 3.4. Quasi-Real Engineering Verification Based on Open Datasets

To further address the practical applicability of the proposed model, an open-data-driven quasi-real verification was conducted using California Department of Transportation Performance Measurement System (Caltrans PeMS) District 8 station 5-min traffic data [15]. The PeMS dataset provides measured traffic flow, average speed, and observation-quality information for freeway monitoring stations. The validation uses the available 2024 records from 1 January to 20 January, covering 5760 consecutive 5-min timestamps. To keep the quasi-real verification internally consistent, this study uses a single traffic dataset with unified spatial coverage, sampling interval, and observation standard rather than combining independent datasets from different locations.

Eight mainline stations on the same freeway and travel direction were selected as measured road-area units. The automatic selection retained only ML stations with complete time coverage and 100% valid observations, and selected stations on I-15 southbound to represent multiple traffic-driven units within one freeway corridor. For each station, the traffic-driven equivalent service output was constructed from total flow and normalized average speed. The 95th percentile of this measured output was used as the equivalent rated capacity for calculating capacity weights. Because PeMS does not provide hardware failure records for the energy units themselves, the individual health values $A_{i}$ follow Table 4 so that the validation focuses on measured spatiotemporal coupling rather than equipment failure modeling.

Each evaluation window contains T = 288 samples, corresponding to a 24 h period at 5-min resolution, and slides forward by 72 samples, corresponding to 6 h. The maximum alignment lag is set to K = 24 samples, corresponding to 2 h, satisfying K/T = 0.083 < 0.1. The same β = 3.0 is used. After normalization into a common service dimension, $\gamma_{ij}$ is set to 1 and $\eta_{ij}$ = $w_{i}$ $w_{j}$ is used to calculate weighted pairwise synergy. The resulting capacity-weighted health baseline is $B_{w}$ = 0.911. A mean zero-lag Pearson coherence of the eight measured output sequences is also calculated as a reference indicator.

A total of 77 continuous 24-h windows were evaluated. Representative high-, medium-, and relatively low-coupling windows were selected according to the calculated regional availability, as shown in Table 5.

The results demonstrate that the proposed index can be calculated from measured traffic-driven flow-speed sequences. When the reference coherence and weighted synergistic potential are high, and the pairwise dispersion is small, $A_{region}$ reaches 0.846. As the measured-output coherence weakens and $\sigma_{w}$ increases, $C_{w}$ decreases from 0.983 to 0.737 and $A_{region}$ decreases to 0.594. This trend is consistent with the numerical scenarios while providing additional evidence that the model can differentiate practical coupling states beyond idealized sine-wave simulations.

## 4. Conclusions

In this paper, a regional availability evaluation model tailored for road-area high-entropy energy based on synergy factors is proposed, which decomposes regional availability into a multiplicative product of the capacity-weighted health baseline, weighted temporal synergy, and weighted spatial consistency coefficient. Specifically, the health baseline is characterized by the capacity-weighted mean of individual unit availabilities. The temporal synergy, anchored on the best alignment similarity that accounts for phase differences, utilizes an exponential saturation function to depict the nonlinear characteristics of synergistic gains. Meanwhile, the spatial consistency coefficient is normalized via the weighted coefficient of variation, serving as a metric to reflect the uniformity of the synergy distribution and overall system robustness. Capacity weights and pairwise coupling coefficients extend the model from equal-capacity isomorphic units to heterogeneous road-area energy units. The mathematical effectiveness of the proposed framework is validated through numerical case studies, and an open-data-driven quasi-real engineering verification using measured Caltrans PeMS traffic-flow records further demonstrates that the model can process practical time-series inputs and differentiate high-, medium-, and relatively low-coupling operating states. Furthermore, parameter sensitivity analysis reveals that the computational results maintain good robustness when *β* fluctuates within the recommended interval of [2.5, 3.5], while variations in the safety factor have limited impact on the final outcomes.

## Figures and Tables

**Table 1:** Unit Availability Values.

Unit Number	1	2	3	4	5	6	7	8
$P_{i}^{rated}$ (kW-equivalent)	60	180	80	140	200	70	160	110
$w_{i}$	0.060	0.180	0.080	0.140	0.200	0.070	0.160	0.110
$A_{i}$	0.92	0.88	0.95	0.90	0.87	0.93	0.89	0.91

**Table 2:** Spatial Consistency Coefficients Across Scenarios.

**Scenario**	$\mu_{w}$	$\sigma_{w}$	$C_{w}$
S1	0.998	0.006	0.994
S2	0.848	0.005	0.994
S3	0.083	0.014	0.855

**Table 3:** Regional Availability Across Scenarios.

**Scenario**	***B_w_***	***S_w_*** **(*t*)**	***C_w_***	$A_{\mathrm{region}}$
S1	0.897	0.950	0.994	0.847
S2	0.897	0.922	0.994	0.822
S3	0.897	0.221	0.855	0.170

**Table 4:** Open-data-based engineering validation settings.

Item	Configuration	Use in the Model
Open dataset	Caltrans PeMS District 8 station 5-min data, 1 January 2024 to 20 January 2024	Measured road-traffic excitation from real freeway monitoring stations
Energy-unit setting	N = 8; I-15 southbound ML stations 801291, 817385, 817760, 817762, 822027, 823715, 823813, and 826055	Eight measured traffic-driven road-area units with complete observations
Equivalent capacity	$P_{i}^{rated}$ is the 95th percentile of measured flow-speed equivalent output; $A_{i}$ follows Table 1	Capacity-weighted health baseline $B_{w}$ = 0.911
Evaluation window	T = 288 samples, sliding step = 72 samples, K = 24 samples	Best alignment similarity with K/T = 0.083
Pairwise weighting	$\gamma_{ij}$ = 1 after common-dimension normalization; $\eta_{ij}=w_{i}\;w_{j}$	$\mathrm{Weighted}\;\rho_{w}$ $,\;\sigma_{w}$ $,\;S_{w}\left(t\right),$ $\;C_{w}$ $,\;\mathrm{and}\;A_{region}$
Reference indicator	Mean zero-lag Pearson coherence among the eight output sequences	Supplementary trend check for measured-output consistency

**Table 5:** Open-data-based quasi-real engineering validation results.

Case or Time Period	High-Coupling PeMS Window (7 January 2024 00:00 to 7 January 2024 23:55)	Medium-Coupling PeMS Window (2 January 2024 18:00 to 3 January 2024 17:55)	Relatively Low-Coupling PeMS Window (19 January 2024 00:00 to 19 January 2024 23:55)
$B_{w}$	0.911	0.911	0.911
$\rho_{w}$	0.963	0.815	0.720
$\sigma_{w}$	0.016	0.115	0.256
$S_{w}\left(t\right)$	0.944	0.913	0.885
$C_{w}$	0.983	0.876	0.737
$A_{region}$	0.846	0.729	0.594
Reference coherence	0.949	0.781	0.684
Interpretation	The measured traffic-driven outputs remain highly coordinated; temporal synergy and spatial consistency are both close to 1.	The average synergistic potential remains moderate, and larger pairwise dispersion reduces the spatial consistency coefficient.	The reference coherence and $\rho_{w}$ both decrease, while $\sigma_{w}$ increases; the model correspondingly lowers regional availability.

## Data Availability

All data in this paper are sourced from the open-source PEMS dataset. It is available on the official website at “https://pems.dot.ca.gov/”.
